# Applying a muscle fatigue model when optimizing load-sharing between muscles for short-duration high-intensity exercise: A preliminary study

**DOI:** 10.3389/fphys.2023.1167748

**Published:** 2023-04-24

**Authors:** Florian Michaud, Laura A. Frey-Law, Urbano Lugrís, Lucía Cuadrado, Jesús Figueroa-Rodríguez, Javier Cuadrado

**Affiliations:** ^1^ Laboratory of Mechanical Engineering, Campus Industrial de Ferrol, Universidade da Coruña, Ferrol, Spain; ^2^ Department of Physical Therapy and Rehabilitation Science, University of Iowa, Iowa City, IA, United Sates; ^3^ Department of Physical Medicine and Rehabilitation, University Hospital Complex, Santiago de Compostela, Spain

**Keywords:** muscle force, multibody dynamics, injury prevention, sport performance, muscle fatigue model, musculotendon model, musculotendon dynamic, ergonomics

## Abstract

**Introduction:** Multiple different mathematical models have been developed to represent muscle force, to represent multiple muscles in the musculoskeletal system, and to represent muscle fatigue. However, incorporating these different models together to describe the behavior of a high-intensity exercise has not been well described.

**Methods:** In this work, we adapted the three-compartment controller (3CCr) muscle fatigue model to be implemented with an inverse-dynamics based optimization algorithm for the muscle recruitment problem for 7 elbow muscles to model a benchmark case: elbow flexion/extension moments. We highlight the difficulties in achieving an accurate subject-specific approach for this multi-level modeling problem, considering different muscular models, compared with experimental measurements. Both an isometric effort and a dynamic bicep curl were considered, where muscle activity and resting periods were simulated to obtain the fatigue behavior. Muscle parameter correction, scaling and calibration are addressed in this study. Moreover, fiber-type recruitment hierarchy in force generation was added to the optimization problem, thus offering an additional novel muscle modeling criterion.

**Results:** It was observed that: i) the results were most accurate for the static case; ii) insufficient torque was predicted by the model at some time points for the dynamic case, which benefitted from a more precise calibration of muscle parameters; iii) modeling the effects of muscular potentiation may be important; and iv) for this multilevel model approach, the 3CCr model had to be modified to avoid reaching situations of unrealistic constant fatigue in high intensity exercise-resting cycles.

**Discussion:** All the methods yield reasonable estimations, but the complexity of obtaining accurate subject-specific human models is highlighted in this study. The proposed novel muscle modeling and force recruitment criterion, which consider the muscular fiber-type distinction, show interesting preliminary results.

## 1 Introduction

Computer modeling and simulation of muscle forces is a well-studied topic since Hill’s early models over 50 years ago (Hoy et al., 1990). These simulations provide useful substitutive approaches to estimate muscular forces (Michaud et al., 2021), (Lamas et al., 2022) during human activities because of the invasive character of *in vivo* experimental measurements, challenges obtaining surface muscle electromyography (EMG) for many deep muscles, and the sometimes inconsistent relation between muscle force and electromyography (EMG) (Dideriksen et al., 2010). Because the muscular system contains redundancies, and is thus an overdetermined system, there have been many attempts to estimate individual muscle contributions through optimized load-sharing paradigms (Michaud et al., 2021). Thus, it is not a novel paradigm to incorporate muscle force models with models of load-sharing. However, as muscles are activated, their force capability is dynamic: declining with continued use due to localized muscle fatigue.

To date, there has been little work done to consider muscle fatigue when applying these mathematical muscle models. For low-force or infrequent muscle activations, this omission is not critical. However, for tasks involving high intensities where loss of muscle force may be expected, the ability to combine muscle fatigue models with muscle force and load-sharing paradigms is increasingly important. These type of simulations may be useful for functional electrical stimulation (FES) (Romero-Sánchez et al., 2019), motor control and prediction (Thelen et al., 2003), or ergonomic applications in which estimates of muscle force over time is relevant, such as may be needed for rehabilitation, prevention of injuries in sports or workplaces, or even surgical planning to reconstruct diseased joints.

Muscle fatigue cannot be modeled as a single universal mechanism, since it follows non-linear behavior, is task-related, and can vary across muscles and joints (Enoka and Duchateau, 2008). In the past few decades, several empirical and theoretical approaches have been proposed to predict the fatigue state of a muscle, or set of agonist muscles, from a given developed force history (Ma et al., 2009), (Xia and Frey Law, 2008), (Liu et al., 2002), but these models have been validated thus far predominantly with known, constant or very simple target loads (TLs). Validation relative to complex movements and muscle contractions is often challenging as the underlying muscle forces can be difficult to ascertain.

Several fatigue modeling approaches have been proposed in the literature. Ding, Wexler, and colleagues utilized a mathematical model of muscle force and added a decay coefficient, but this model was primarily of use for electrically-activated muscle (Ding et al., 2000), (Ding et al., 2003). Ma et al. (2009) developed a muscle fatigue model that incorporates multiple parameters and has shown promise, but is not as clearly able to be combined with a Hill muscle model. Xia and Frey-Law developed a three-compartment controller (3CC) model to enable it to handle any time-varying force profile using a feedback controller to match target loads (TLs) either at a single muscle or joint-level (Looft et al., 2018). This 3CC model was an improvement over a similar earlier model that could only represent maximum activation (Liu et al., 2002). The 3CC approach has been evaluated, validated and modified (giving place to the 3CCr model that uses an additional rest recovery parameter) by the same and other investigators including Looft et al. (2018), Sonne and Potvin. (2016), Barman et al. (2022) who applied it at the joint-level for fatigue prediction of various isometric tasks and posture optimization. Thus, this fatigue model provides a relatively simple means that can be applied in multiple formats depending on the application.

Relatively little work has combined multilevel models considering the redundant muscle forces within a multibody environment with muscle fatigue. One recent example was by Pereira et al. (2011) who presented a methodology to include a muscle fatigue model that allows the calculation of muscle force redundancies. However, they did not apply the resulting model to a real case, and, consequently, did not address numerous potential issues that can come up when dealing with actual real-world tasks.

Mode accuracy depends on both the underlying equation formulation (i.e., non-linear or exponential behavior) as well as the defining model parameter values. Thus, the determination of these model parameters is a critical aspect of model development. Some modeling approaches use average behavior to define model parameters (Frey-Law et al., 2021), which may be applicable for making general conclusions for population-level concerns, such as workplace ergonomic issues. However, when representing individual responses to tasks, it becomes necessary to calibrate subject-specific parameters. Traditional muscle force parameters explicitly depend on the musculoskeletal geometry (e.g., musculotendon length, musculotendon shortening velocity, and moment arms), while others can be indirectly scaled (e.g., based on optimal muscle fiber length and slack tendon length). Depending on the modeling approach, maximum isometric force as well as fatigue and recovery coefficients require additional calibration measurements. All these parameters play essential roles in an accurate dynamic force-time simulation as they each are known contributors to muscle force generation.

For this reason, the aim of this preliminary investigation was to assess the methodology required to produce time-varying muscle force predictions for a high-intensity dynamic task, through the combination of three previously described, distinct modeling approaches into one comprehensive subject-specific multi-level muscle model. We consider model permutations using several options: single versus multiple muscle representation of the elbow joint (Michaud et al., 2021), (Xia and Frey Law, 2008) and also the addition of muscle fiber-type recruitment order (Henneman et al., 1965). We present a benchmark case: the force quantification of the elbow flexor and extensor muscles involved in static isometric flexion and a dynamic weightlifting task (i.e., the hammer curl) involving cycles of forearm flexion and extension, using data collected from a single example subject for comparison. We present several practical concerns that arise when modeling muscle force in this way, offering strategies for others.

## 2 Material and methods

### 2.1 Experimental data collection

**FIGURE 1:**
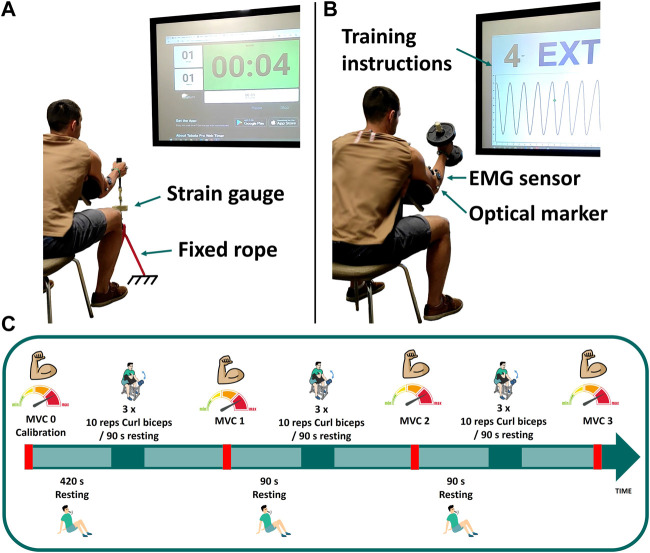
Experimental measurements: **(A)** maximum voluntary contraction; **(B)** dumbbell weightlifting; **(C)** schematic of the test protocol for visit 1.

One subject (male, age 31, height 180 cm, body mass 80 kg) was recruited for this study involving two study visits. He provided written informed consent as approved by the Committee of Ethics of the University of A Coruña prior to all participation. He was asked to perform dynamic (at visit 1) and static (visit 2) elbow flexion tasks while motion capture (18 optical infrared cameras OptiTrack FLEX 3 sampling at 100 Hz; Natural Point, Corvallis, OR, United States) and muscle electromyography (EMG, FREEEMG sampling at 1,000 Hz, BTS, Quincy, MA, United States) were assessed. In order to isolate the activity to the elbow joint, the subject was seated in a chair, with his elbow secured to an armrest to minimize force generation from other joints (Figure 1). Before commencing the elbow flexion tasks, the subject completed a 10-min warm-up using a resistance band and carried out a series of the exercises at a submaximal level to minimize risk of injury, optimize muscle potentiation, and ensure that the sensors attached to his body did not hinder his performance.

#### 2.1.1 MVC0: Calibration

At the first visit, three isometric maximum voluntary contractions (MVCs) were assessed at baseline (MVC0) to be used for EMG normalization as well as model parameter identification. These elbow flexion MVCs were assessed with his elbow positioned at 50° of flexion by pulling on a fixed rope in series with a strain gauge (Phidgets Micro Load Cell 0–20 kg, sampling at 100 Hz) (Figure 1A). Three repetitions were collected: first sustained for 8 s (MVC0-1), followed by a 15 s rest; the second sustained until failure (MVC0-2), again with a 15 s rest; and the third sustained for 6 s (MVC0-3). EMG measurements recorded during MVC0-1 were used to normalize the EMG signal. Then, only MVC0-2 and MVC0-3 were used to calibrate the fatigue and force parameters and to select the weight of the dumbbell, in order to avoid possible errors introduced by muscle potentiation (Lorenz, 2011; Blazevich and Babault, 2019) after MVC0-1. The MVC0 calibration session was followed by a resting period of at least 7 min to facilitate complete recovery (BRODY, 1999) before beginning the dynamic test.

#### 2.1.2 Dynamic effort: Training session

The dynamic task was performed on day 1, consisting of 3 cycles of 3 sets of 10 repetitions of hammer curls, with the hand in a neutral pronation/supination position when holding the dumbbell (see Figure 1). The dumbbell weight was chosen to correspond to 65% of the subject’s measured maximal force (MVC0-1). Each set was followed by a resting period of 90 s, and MVCs were repeated after each cycle (MVC1, MVC2 and MVC3). For these MVC captures, the subject was asked to pull the fixed rope 3 times for 8 s each, with 6 s resting periods between each repetition. Instructions (number of repetitions, rate, rest periods, etc.) for each task were provided verbally and visually, displayed on a big screen situated in front of the subject (Figure 1).

The entire session was simulated using the full muscle model, including the weightlifting series, the resting periods and the MVC evaluations.

#### 2.1.3 Static effort

At the second visit, a few days later to avoid injury and fatigue accumulation, the subject returned and was asked to perform the static task. For this visit, he was asked to hold the same dumbbell weight used for the dynamic task while maintaining the arm flexed at 50° until failure. Thus the static task was a submaximal effort. The MVCs were not repeated, thus the model parameters calibrated using the MVC0 repetitions (performed at visit 1) were also used for the static effort simulation.

#### 2.1.4 Motion capture and EMG

The motion was captured using 18 optical infrared cameras (OptiTrack FLEX 3, also sampling at 100 Hz; Natural Point, Corvallis, OR, United States) that computed the position of 9 optical markers for the trunk and right arm, and 2 additional markers fixed on the strain gauge to monitor the force orientation. Additionally, 3 surface EMG sensors from the right arm (biceps long head, biceps short head and brachioradialis) were recorded at 1 kHz (BTS, FREEEMG, Quincy, MA, United States). The electrodes were placed according to the guideline presented in (Criswell and Cram, 2011). Each EMG signal was rectified and filtered by singular spectrum analysis (SSA) with a window length of 250 msec (Romero et al., 2016) (equivalent to a forward and reverse low-pass fifth order Butterworth filter with a cut-off frequency of 6 Hz). Then, because muscle fatigue affects the amplitude of the surface EMG (Dideriksen et al., 2010), the filtered EMG signals were normalized using the maximal value observed during the subject’s first MVC.

### 2.2 Musculoskeletal models

**FIGURE 2:**
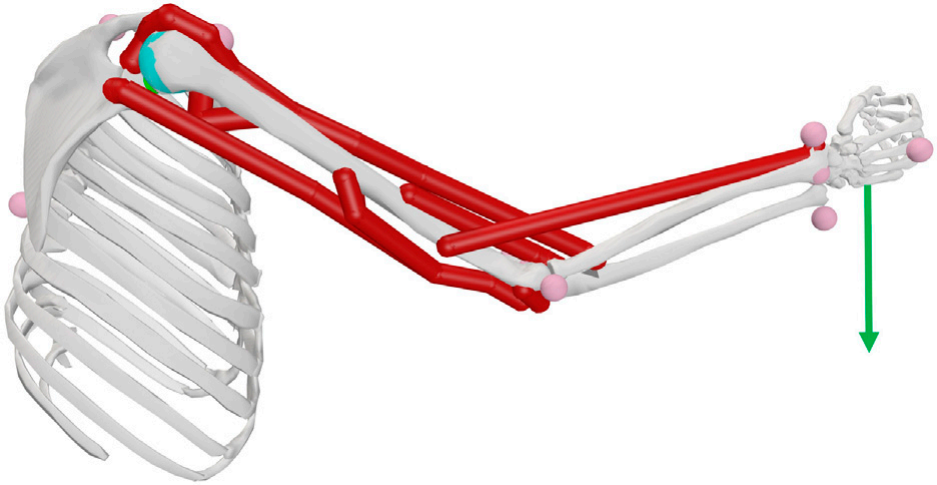
Upper right extremity model including the elbow joint with seven muscle actuators represented. Note the external force is represented by the green arrow applied at the hand center of mass (COM).

Three right upper extremity musculoskeletal model representations were considered. The primary model (Figure 2) included 7 muscles (triceps long, medial and lateral head, biceps long and short head, brachioradialis and brachialis) was adapted from (Saul et al., 2015), with link lengths scaled to the subject. Using this 7-muscle arm model (7M), inverse dynamic analyses were used to determine elbow torques (*Q*), assuming the rope or dumbbell acted as external forces applied to the right hand center of mass (see green arrow in Figure 2). A simpler (2-muscle) and more complex (addition of fiber type) version of this base 7M musculoskeletal model were also considered, and are described later in Sections 2.6.1 and 2.6.2 respectively.

### 2.3 Muscle coordination strategies and musculotendon model

Determination of muscle forces by computer modeling and simulation is challenging due to the redundancy in the biomechanical system. Numerous approaches can be found in the literature to solve the problem of identifying muscle recruitment levels, as well as to represent the musculotendon actuator dynamics (Michaud et al., 2021), (Lamas et al., 2022). The fundamental problem is that there are more muscles serving each degree of freedom of the system than those strictly necessary from the mechanical point of view. In this case, there are 7 muscles acting about the elbow joint to actuate a single degree of freedom, either flexion or extension. Other degrees of freedom of the elbow are controlled by joint structures as bones and ligaments, yielding a reaction moment instead of a drive torque. Consequently, there is an infinite number of solutions for this problem, and, in order to reproduce the specific strategy of muscle coordination adopted by the central nervous system (CNS), optimization is often used.

The muscular forces for each of the 7 muscles are identified using a combination of optimization load-sharing (Eq. 1) and the net joint torque formulated in general form as Eq. 2 at each time-point of the tasks (Michaud, 2020).

$$\begin{array}{ll} \min\;C_{f} & \\ subject\;to & J^{T}F^{MT}=Q\\ & F_{i}^{\mathrm{Min}}\leq F_{i}^{MT}\leq F_{i}^{\mathrm{Max}}\;i=1,2,...,m \end{array}$$ (1)

where $C_{f}$ is the cost function, Q is the vector of joint torques obtained by inverse dynamics, $F^{MT}$ is the vector of the individual muscle forces, J is the Jacobian whose transpose projects the muscle forces into the joint drive torques space (i.e., represents the moment arms), $F_{i}^{\mathrm{Min}}$ ; $F_{i}^{\mathrm{Max}}$ are the instantaneous minimum and maximum allowed forces for muscle *i*, respectively, and *m* is the number of muscles (m = 7). Note that in this particular case, the elbow joint involves 1 plane of muscle flexion/extension torques, thus, Q simplifies to a scalar. The expression of the objective function $C_{f}$ depends on the muscle recruitment criterion used. Several muscle recruitment criteria have been suggested, typically consisting of sums of muscle forces divided by a positive weighting factor (usually the corresponding maximum isometric force or the physiologic cross sectional area) to a power of 2 or more, to best represent empirical CNS behavior (Michaud et al., 2021). We chose the minimization of the sum of the squares of muscle forces (Eq. 1) based on the comparisons provided by Michaud and colleagues (Michaud et al., 2021).

$$C_{f}={\sum_{i=1}^{m}\left(F_{i}^{MT}\right)}^{2}$$ (2)

**FIGURE 3:**
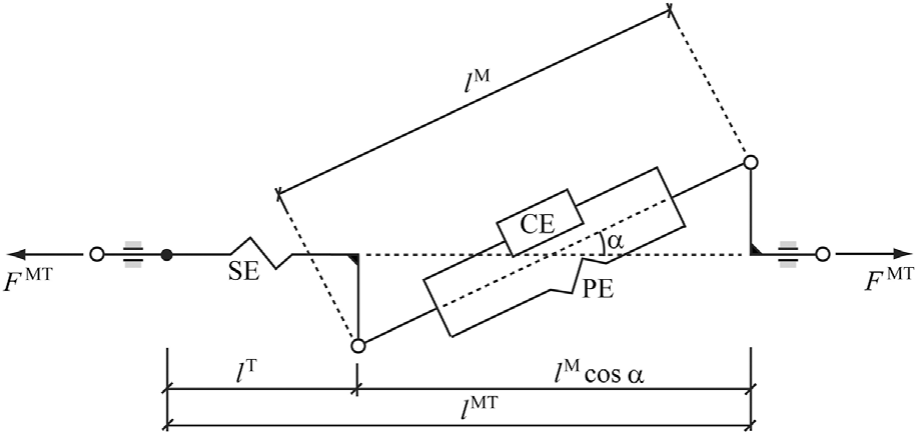
Hill-type musculotendon model. The muscle fibers are modeled as an active contractile element (CE) in parallel with a passive elastic component (PE). These elements are in series with a non-linear elastic tendon (SE). The pennation angle *α* denotes the angle between the muscle fibers and the tendon. Superscripts MT, M, and T indicate musculotendon, muscle fiber, and tendon, respectively.

In this work, the so-called physiological approach was implemented to prescribe the minimal and maximal constraints (which affect the fatigue behavior) for the forces through feasible muscle dynamics (Michaud et al., 2021). The force generated by a muscle depends on its force-length-velocity properties, as well as its physiologic cross sectional area (PCSA), and is related to the Hill-type musculotendon model (Figure 3) used (Zajac, 1989), where the force equilibrium equation for a muscle is provided as Eq. 3:

$$F^{MT}=\left(F_{CE}^{M}+F_{PE}^{M}\right)\cos\alpha$$ (3)

In this equation, $F_{CE}^{M}$ and $F_{PE}^{M}$ are the forces exerted by the contractile element (CE) and the passive element (PE), respectively, and α is the pennation angle. The active force produce by CE depends on the muscle fiber length and velocity, and on the activation level. It is expressed as shown in Eq. 4:

$$F_{CE}^{M}=F_{0}^{M}\times a\times f_{l}\left(l^{M}\right)\times f_{v}\left(v^{M}\right)$$ (4)

where a represents the muscle activation, $l^{M}$ the muscle fiber length and $v^{M}$ its velocity, $f_{l}$ and $f_{v}$ are dimensionless force-length and force-velocity relationships, respectively and $F_{0}^{M}$ represents the force magnitude proportional to cross-sectional area (Michaud, 2020). In this work, the tendon length is considered constant (rigid tendon assumption) and, consequently, the muscle fiber length and velocity only depend on the musculoskeletal geometry and the motion of the segments, and not on the musculotendon force (Michaud et al., 2021). Moreover, the muscular time response to the excitation is ignored, thus assuming that a=u , where u is the excitation. In Figure 3, $l^{MT}$ is the musculotendon length and $v^{MT}$ is the musculotendon velocity.

The force of the parallel passive element, which opposes muscle stretch, $F_{PE}^{M}$ , can be formulated as Eq. 5

$$F_{PE}^{M}=F_{0}^{M}\times f_{PE}\left(l^{M}\right)$$ (5)

where $f_{PE}$ is a dimensionless force-length relationship, which has non-zero value when muscle length is greater than the optimal muscle fiber length ( $l_{0}^{M}$ ).

The instantaneous minimum and maximum allowed forces in muscle *i*, $F_{i}^{\mathrm{Min}}$ and $F_{i}^{\mathrm{Max}}$ , were calculated using $a_{i}$ = 0 (no active contraction) and $a_{i}$ = 1 (maximal active contraction), respectively. In this way, by combining Eqs 3–5 the resulting minimum and maximum single muscle forces are represented by Eqs. 6, 7, respectively.

$$F^{\mathrm{Min}}=f_{PE}\left(l^{M}\right)\times F_{0}^{M}\times \cos\alpha$$ (6)

$$F^{\mathrm{Max}}=\left(f_{l}\left(l^{M}\right)\times f_{v}\left(v^{M}\right)+f_{PE}\left(l^{M}\right)\right)F_{0}^{M}\times \cos\alpha$$ (7)

After finding $F^{MT}$ from optimization (*fmincon*, Matlab), the corresponding muscle activation, *a*, was determined from the following equation:

$$a=\left(\frac{F^{MT}}{\cos\alpha }-F_{PE}^{M}\right)/F_{CE,\mathrm{Max}}^{M}$$ (8)

where $F_{CE,\mathrm{Max}}^{M}$ corresponds to the maximum contractile force ( a = 1).

### 2.4 Subject-specific scaling or calibration of musculotendon parameters

A table that summarizes the different subject-specific scaling or calibration of musculotendon parameters reported in the following sections can be found in Supplementary Appendix SA1 for better understanding and comparison.

#### 2.4.1 Scaling of length parameters

Due to the sensitivity of physiological approaches (Scovil and Ronsky, 2006), a suitable scaling of musculotendon parameters is needed. Hill-muscle equations become numerically stiff when numerical singularities are approached (Millard et al., 2013), which often occurs during a simulation, resulting in the solver finding solutions that are numerically feasible yet not physiologically sound (Van Campen et al., 2014). To prevent this problem, the scaling correction applied in (Michaud et al., 2021) was reproduced in this work to scale the tendon slack length ( $l_{S}^{T}$ ) and the optimal muscle fiber length ( $l_{0}^{M}$ ), which affect the dimensionless force-length function. Muscle properties and local coordinates for the attachments of muscle and tendon to bone were obtained from OpenSim (DAS model) and scaled to each subject. $l_{S}^{T}$ and $l_{0}^{M}$ were scaled, for each muscle, with a scale factor calculated as the relation between the subject’s musculotendon length with outstretched arm and that of the OpenSim model in the same position.

#### 2.4.2 Calibration of moment arms

The moment arms of the muscles in the model obtained by the open source model (Saul et al., 2015) showed inconsistencies when estimating elbow flexion muscle forces. That is flexion torque during the hammer curl was much higher than those estimated during the MVC and, hence, needed to be modified because the wrong moment arms conditioned the exerted force and, consequently, the corresponding fatigue. First, the brachioradialis insertion points (which showed the most unrealistic geometry) were corrected from a more recent OpenSim dynamic arm simulator (DAS) model (Chadwick et al., 2014). Second, a scale factor was calculated to reduce the variations produced in isometric strength as reported below.

During the maximum voluntary contraction of flexors, the maximum inverse-dynamics elbow joint flexion moment $Q_{\mathrm{Max},Flex}$ at the right arm can be defined as:

$$Q_{\mathrm{Max},Flex}\left(t\right)=\left[\begin{array}{cc} J_{Flex}^{T}\left(t\right) & J_{Ext}^{T}\left(t\right) \end{array}\right]\left[\begin{array}{c} F_{Phys,Flex}^{\mathrm{Max}}\left(t\right)\\ F_{Phys,Ext}^{\mathrm{Min}}\left(t\right) \end{array}\right]$$ (9)

where flexors were considered to be producing their maximum ( a = 1) allowed force ( $F_{Phys,Flex}^{\mathrm{Max}}$ ) during the motion, and extensors their minimum ( a = 0, co-contraction not considered, only passive force) allowed force ( $F_{Phys,Ext}^{\mathrm{Min}}$ ). Rearranging (Eq. 9), it can be written,

$$J_{Flex}^{T}\left(t\right)F_{Phys,Flex}^{\mathrm{Max}}\left(t\right)=Q_{\mathrm{Max},Flex}\left(t\right)-J_{Ext}^{T}\left(t\right)F_{Phys,Ext}^{\mathrm{Min}}\left(t\right)$$ (10)

with $J_{Flex}^{T}$ and $J_{Ext}^{T}$ the Jacobians whose transposes project the muscle forces into the joint drive torques space of flexor and extensor muscles, respectively. To simplify, (Eq. 10) can be expressed as

$$J_{Flex}^{T}\left(t\right)F_{Phys,Flex}^{\mathrm{Max}}\left(t\right)=Q_{\mathrm{Max},Flex}^{*}\left(t\right)$$ (11)

where $Q_{\mathrm{Max},Flex}^{*}$ is the maximum inverse-dynamics elbow joint flexion moment taking into account the passive moment generated by the extensor muscles.

To continue, the problem was reduced to a single scale parameter for calibration by considering the flexor muscles as a single actuator (Lamas et al., 2022). In this way, by combining (Eqs 5, 6, 8, 11), it is obtained,

$$J_{Flex}\left(t\right)\left(F_{0}^{M}\times a\left(t\right)\times f_{l}\left(t\right)\times f_{v}\left(t\right)+F_{0}^{M}\times f_{PE}\left(t\right)\right)\cos\alpha \left(t\right)=Q_{\mathrm{Max},Flex}^{*}\left(t\right)$$ (12)

The moment arm, $J_{Flex}$ , the dimensionless force-length function, *f*_*l*,_ and the dimensionless force-velocity function, *f*_*v*_, of the actuator, were calculated as the average of the corresponding values of the flexor muscles during the motion. The pennation angle, α , was set to 0° to simplify, and, as mentioned previously, the activation, *a*, was set to 1 during MVC. Therefore, if the moment arms were accurate, the extracted maximum isometric force of the corresponding actuator, $F_{0}^{M}$ , should be the same at any position of the arm, and could be expressed as:

$$F_{0}^{M}=Q_{\mathrm{Max},Flex}^{ID*}\left(t\right)/\left(J_{Flex}\left(t\right)\left(f_{l}\left(t\right)\times f_{v}\left(t\right)+f_{PE}\left(t\right)\right)\right)$$ (13)

However, by estimating the maximum force of the actuator during MVC at four different positions (three maximum efforts per position, but considering only the last two to avoid muscle potentiation effects) of elbow flexion (from 15° to 60°), large differences were observed between the values (Table 1). For example, the estimated force at position 1 (15° flexion) was 160% higher than the estimated force at position 4 (60° flexion).

**TABLE 1:** Maximum actuator force (condensed maximum isometric force) estimated at different arm flexion angles for moment arm calibration.

		Maximum actuator force (N)									
		Pos 1		Pos 2		Pos 3		Pos 4		Mean	SD
Original	Static	1909.0	1852.9	1421.3	1412.7	1338.5	1313.9	1127.9	1179.7	1444.5	288.5
	Phys	1912.4	1857.9	1426.1	1415.8	1339.0	1309.5	1165.6	1203.9	1453.8	281.7
Optimized	Phys	1957.3	1882.8	1865.9	1774.1	1853.7	1809.7	1892.4	1916.1	1869.0	58.0

Note that, to minimize fatigue effects, the MVC assessment used for moment arm calibration was conducted on a different day than the other experimental measurements, with 7 min resting periods between MVCs to enhance recovery between trials (BRODY, 1999). In addition, in order to rule out the possible effect of the dimensionless force-length and force-velocity functions, the maximum force of the actuator was also estimated without considering the physiological approach ( $F_{0}^{M}=Q_{\mathrm{Max},Flex}\left(t\right)/J_{Flex}\left(t\right)$ ), showing almost the same results (Table 1, Static).

To overcome the moment arms inconsistencies problem, two scale factors ( $k_{1}$ ) was calculated to reduce the variations produced in the isometric force by the average moment arm, $J_{Flex}\left(\varphi \right)$ , with φ the elbow flexion angle. The calibrated relation $J_{Flex}^{*}\left(\varphi \right)$ is given by:

$$J_{Flex}^{*}\left(\varphi \right)=k_{1}\left(J_{Flex}\left(\varphi \right)-J_{Flex}\left(0{}^{\circ}\right)\right)+\frac{J_{Flex}\left(0{}^{\circ}\right)}{k_{1}}$$ (14)

where $k_{1}$ is the optimized value that minimizes the standard deviation (SD) between the eight forces estimated at the four positions (*fmincon*, Matlab). As shown in Table 1, the difference between the maximum actuator forces at positions 1 and 4 was reduced to 1%, and the standard deviation was almost divided by a factor of 5.

The optimized coefficient $k_{1}$ which reduced the moment arm differences during the motion was applied to the moment arm of each muscle. The same procedure could be replicated for the extensors or for other joints by isolating the desired effort. However, it must be said that this calibration would not be necessary if the musculoskeletal model was correct. Finally, an alternative way to correct muscle geometry would be to move the origin and insertion points of the muscles, but this method would have involved a larger optimization problem.

#### 2.4.3 Calibration of maximum isometric force

Another subject-specific parameter which is critical for accurate fatigue simulation (because the relative target load of the task depends on it) is the maximum isometric force, $F_{0}^{M}$ . In some studies, this parameter is adapted to allow muscles to produce the calculated joint torques, adding even a reserve (by overincreasing $F_{0}^{M}$ ), or adding residual actuators to the model. These residual actuators are called “the hand of God,” and are forces that account for discrepancies between the model, the measured motions, and the muscular forces that are not able to generate sufficient accelerations (Documentation, 2022). In this study, in addition to affecting muscle recruitment strategy, if the maximum isometric force is overestimated, fatigue will be underestimated and *vice versa*. Therefore, calibration is needed to approximate, as close as possible, force and fatigue limits of the subject (i.e., muscles forces have to be able to produce the calculated joint torques without being underactivated). Only MVC0-2 and MVC0-3 were considered when calibrating the force in order to avoid possible errors introduced by muscle potentiation (Lorenz, 2011; Blazevich and Babault, 2019) after MVC0-1. Starting from Eq. 9) and using the initial $F_{0}^{M}$ given by the model (Saul et al., 2015), the scale parameter of the maximum isometric forces $k_{2}$ can be defined as:

$$k_{2}\left(t\right)=Q\left(t\right)/Q_{\mathrm{Max},Flex}\left(t\right)$$ (15)

And, then, the new scaled maximum isometric forces for all the muscles are modeled as:

$$F_{0}^{M*}=\max\left(k_{2}\right)F_{0}^{M}$$ (16)

In this work, the scale parameter for the arm extensors was taken the same as for the flexors. However, the same procedure could be replicated for the extensors during MVC, focusing on extension.

### 2.5 Muscle fatigue model

Muscle fatigue is a multidimensional concept that combines physiological and psychological aspects, and which is defined as a decrease in maximal force or power production in response to contractile activity (Gandevia, 2001). It can originate at different levels of the motor pathway and is usually divided into central and peripheral components. Central fatigue originates at the CNS, and decreases the neural drive to the muscle (Gandevia, 2001). Peripheral fatigue takes place in the periphery, at the neuromuscular junction and within the muscle involving processes associated with mechanical and cellular changes (WALLMANN, 2007). Although central fatigue can play a major role in muscular performance, it particularly affects low frequency fatigue and endurance sports, and is challenging to model as it can induce changes over several days. For these reasons, this study will be limited to the modeling of peripheral, localized muscle fatigue and to its implementation within the muscle recruitment problem.

**FIGURE 4:**
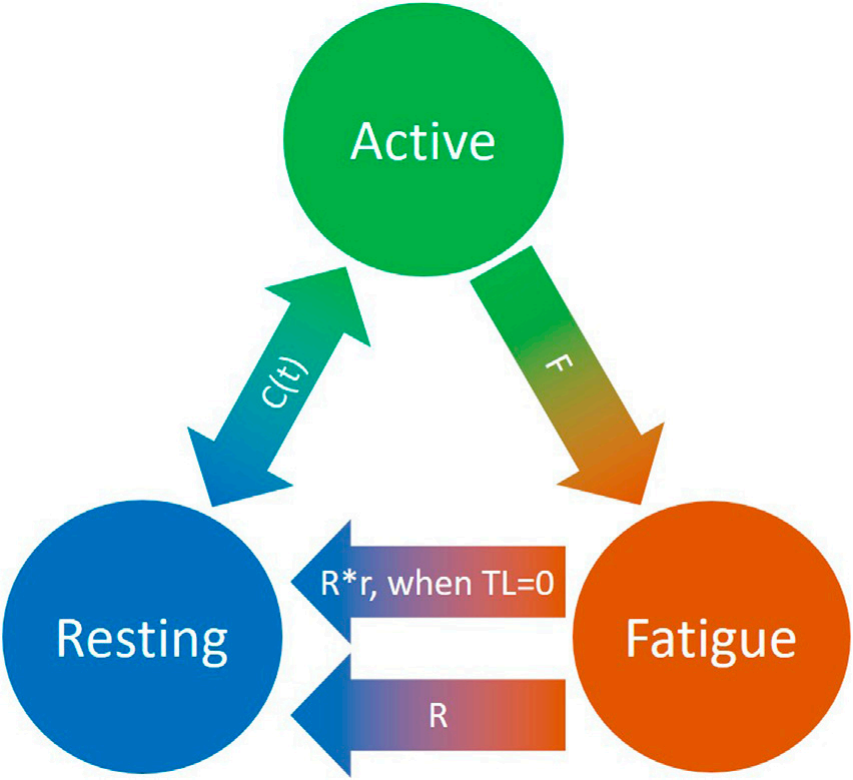
Schematic representation of the revised three-compartment controller (3CCr) mathematical fatigue model reproduced from (Frey-Law et al., 2021) licensed under CC BY-NC-ND 4.0, where C (t) is the feedback controller used to match Ma to target load, TL; F defines fatigue rate and R defines recovery rate, with additional recovery during rest periods signified by r.

To quantitatively evaluate task-related muscle fatigue for complex and/or dynamic movements, we used the three-compartment model (Figure 4), described by Xia and Frey Law (3CC) (Xia and Frey Law, 2008), and later improved (3CCr) in (Looft et al., 2018), (Frey-Law et al., 2021), to describe muscle activation (*M*_*a*_), fatigue (*M*_*f*_), and recovery (*M*_r_) under a variety of loading conditions. The sum of the percentage of motor units (MU) in each compartment equals 100%. While the model is explained in greater detail in (Xia and Frey Law, 2008), a brief summary is provided here. During activity, MUs from the resting compartment are moved into the activated compartment at a rate controlled by a feedback controller, C(t), to match the target load, TL, represented as a percentage of maximum (% of MVC). This controller also allows for the reverse movement of motor units (from *M*_a_ to *M*_r_) if more units are activated than needed to match a certain TL. The flows between the three compartments are mathematically described as differential equations as follows:

$$\frac{dM_{r}}{dt}=-C\left(t\right)+\left(R\times r\right)\times M_{f}$$ (17)

$$\frac{dM_{a}}{dt}=C\left(t\right)-R\times M_{a}$$ (18)

$$\frac{dM_{f}}{dt}=F\times M_{a}-R\times M_{f}$$ (19)

where,

$$C\left(t\right)=\left(TL-M_{a}\right)\;when\;M_{a}<TL\;and\;M_{r}>\left(TL-M_{a}\right)$$ (20a)

$$C\left(t\right)=M_{r}\;when\;M_{a}<TL\;and\;M_{r}<\left(TL-M_{a}\right)$$ (20b)

$$C\left(t\right)=\left(TL-M_{a}\right)\;when\;M_{a}>TL$$ (20c)

r=1 when TL > 0 (20d)

*F* and *R* denote the fatigue and recovery coefficients, respectively, and *r* is a rest multiplier to augment recovery during rest (Looft et al., 2018). While there are normative, joint-specific values identified for these coefficients for average behavior (Xia and Frey Law, 2008), (Looft et al., 2018), they are not subject-specific. For this study, the calibration of *F, R* and *r* is defined in Section 2.6.

**FIGURE 5:**
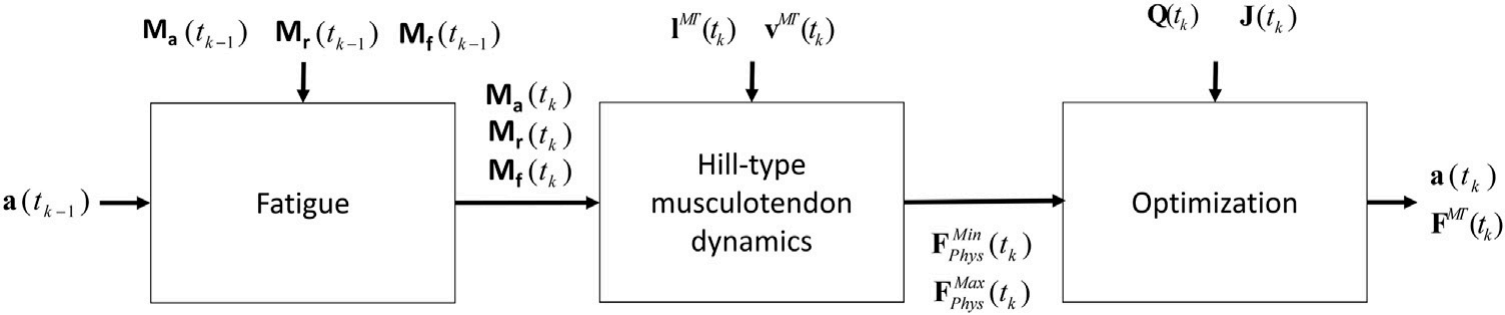
Procedure proposed that combines the physiological inverse-dynamics approach (used to address the muscle redundancy problem) with the muscle fatigue model, to determine individual muscle forces at time instant *t*_*k*_.

Due to the complexity of the muscle redundancy problem, the original model was only used to predict fatigue at joint level in terms of joint torque decay (Xia and Frey Law, 2008), and not to predict the fatigue of the individual muscles in the musculoskeletal model. This was achieved by combining the muscle fatigue model with the optimization approach that addressed the muscle redundancy problem, as illustrated in Figure 5. That is, the muscle activations from the previous time step were used to determine net joint target loads (TLs) for the determination of the 3CCr compartment states during the current time step.

Peripheral fatigue affects the contractile portion of muscle torque production, so that only $F_{CE}^{M}$ (Eq. 4) will be affected by the fatigue level of the muscle, thus decreasing the maximum muscle forces allowed in the muscle redundancy problem. Then, because all the non-fatigued muscle units are recruited, the $F^{\mathrm{Max}}$ of Eq. 6) becomes:

$$F^{\mathrm{Max}}=\left(f_{l}\left(l^{M}\right)\times f_{v}\left(v^{M}\right)\times \left(100-M_{f}\right)/100+f_{PE}\left(l^{M}\right)\right)F_{0}^{M}\times \cos\alpha$$ (21)

### 2.6 Muscular modeling and subject-specific calibration of fatigue parameters

#### 2.6.1 Joint actuators and simple muscles

Because physiological measurement of muscle force and fatigue are conducted at the joint level rather than at the individual muscle level (unless invasive procedures are used), yet, modeling of individual muscles allows for modeling of internal stress and strain that is not possible with joint-level models, both strategies were implemented here for comparison. For joint level analysis, the one flexor and one extensor (2M), using the mean parameters of the corresponding muscles (distinguishing between flexors and extensors), except in the case of $F_{0}^{M}$ , for which the sum was used. And, for individual muscle analysis, the seven muscles of the model (7M) were considered individually to take into account their fatigue behavior within the muscle force-sharing problem.

In both cases, fatigue parameters *F*, *R* and *r* were considered the same for all the muscles and calibrated from recorded activities at joint level by means of optimization (*fmincon*, Matlab), seeking to best fit model and experimental results, similar to that proposed by Frey-Law et al. in (Frey-Law et al., 2021). *F, R* and *r* are the variables of the optimization problem aimed to minimize the residuals between model estimates of decaying MVC (*Ma* during MVC trials) and observed MVCs (force measurements during MVC0-2 and MVC0-3). MVC0-2 measurements showed the best visibility of the force decay (to adjust *F*) because force measurements were very noisy and MVC0-2 lasted a long time period (discrepancies were observed by using shorter time periods). MVC0-3 measurements were needed to calibrate the recovery parameter after an effort (to adjust *R and r*).

#### 2.6.2 Splitting of muscular fiber types

**FIGURE 6:**
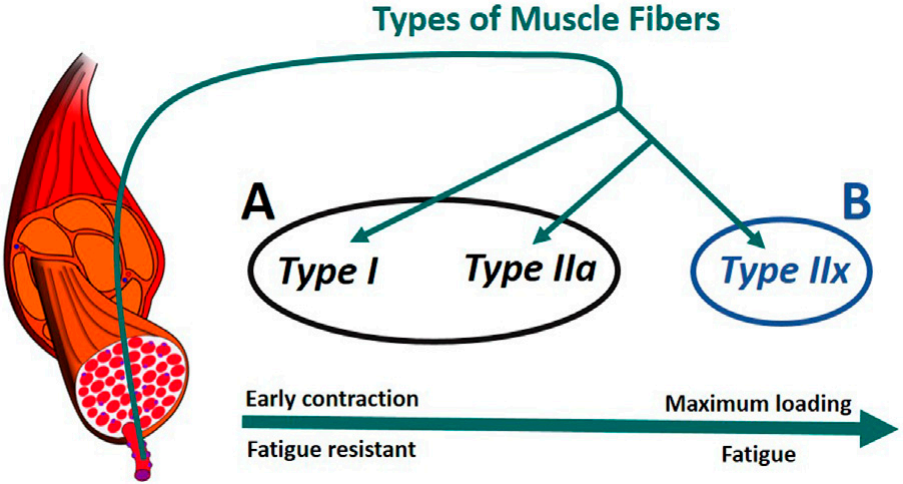
Muscle recruitment hierarchy as function of the fiber (Radák, 2018). **(A)** composed of type I and type IIa fibers. **(B)** formed of type IIx fibers. The muscle fascicles and cells is licensed under Sheldahl, CC BY-SA 4.0, (via Wikimedia Commons).

In humans, there are generally three primary types of skeletal muscle fibers associated with MUs (Figure 6): type I (slow oxidative), type IIa (fast oxidative), and type IIx (fast glycolytic) (Tiedemann et al., 2012). Their characteristics define their properties: i) Type I: large amount of mitochondria, fatigue resistant (hours), low amount of force; ii) Type IIa: moderate amount of mitochondria, moderate amount of glycosomes and creatine kinase, moderate fatigue resistant (<30 min), moderate amount of force; iii) Type IIx: small amount of mitochondria, significant amount of glycosomes, very low fatigue resistant (<1 min), largest amount of force (Betts et al., 2017), (Wilson et al., 2012). In addition, according to Henneman’s size principle (Henneman et al., 1965), there typically exists a recruitment hierarchy in force generation. Slow-twitch fibers (type I) have a low activation threshold, meaning that when a contraction is initiated, they are recruited first, followed by type IIa fibers, and, then, finally, type IIx if needed.

To consider fiber type differences, yet minimize model complexity, we represented muscle as only two groups based on fatigue behavior. Thus we combined type I and type IIa as group A, while type IIx formed group B (Figure 6). Therefore, each muscle in the model was split into two parts, leading to a total of 14 muscle representations (14M) for the muscle recruitment problem. Muscle geometry parameters (moment arm, $l^{MT}$ ; $v^{MT}$ ) remained constant for both groups of each muscle, but all other muscle parameters were adjusted to represent the corresponding fiber type properties in the model.

##### 2.6.2.1 Maximum isometric force distribution

While previous studies identified differences in power between types of isolated fibers (Widrick et al., 2002), the proportion of maximum force due to each type depends on fiber-type distribution of a muscle. This distribution varies by individual, muscle, and physical activity (Wilson et al., 2012), (Picquet et al., 2002), but was simplified as being the same for all 7 muscles for this study, estimated from a calibration measurement conducted at elbow level. Because of the differences in fatigue between the two groups (Betts et al., 2017), we hypothesized that the percentage of force decay (*%decay*) during high intensity exercise (MVC0) may be related to the fiber type force distribution of the subject (not the fiber type distribution itself). Therefore, we considered that almost all the force loss (90% of *%decay*) during this short but very demanding effort, corresponds to group B of fibers (the remaining 10% attributed to group A force decay and group B force reserved). In this way, the maximum isometric forces $F_{0,A}^{M}$ and $F_{0,B}^{M}$ corresponding, respectively, to group A and group B, were defined as:

$$F_{0,A}^{M}={F_{0}^{M}}^{*}\left(100-{\%decay}^{*}0.9\right)$$ (22)

$$F_{0,B}^{M}={F_{0}^{M}}^{*}\left({\%decay}^{*}0.9\right)$$ (23)

##### 2.6.2.2 Fatigue parameters

To represent the fiber properties of group A, their fatigue parameters were set as follows: $F_{A}=0.004$ , $R_{A}=0.01$ and $r_{A}=1$ . These values were set to obtain a reduced fatigue (25% of force decay after 120 s with a *TL* of 100%) and a full force regeneration after 4 min of resting (BRODY, 1999).

To represent the fiber properties of group B, it was decided to fix the recovery coefficient, $R_{B}$ , to 0.001, to represent the anaerobic functioning of cells and their reduced recovery during activation due to the small amount of mitochondria (Betts et al., 2017). $F_{B}$ and $r_{B}$ were obtained by optimization (*fmincon*, Matlab), seeking to best fit model and experimental results from MVC0-2 and MVC0-3. As a preliminary study, these parameter values provided a reasonable initial approach to enable the inclusion of some subject-specific values.

##### 2.6.2.3 Recruitment hierarchy in force generation.

Finally, to reproduce the recruitment hierarchy in force generation, the muscle recruitment criterion (Eq. 2) was modified by adding the fatigue parameter *F* of each muscle as a weight in the objective function, which becomes,

$$C_{f}=\min{\sum_{i=1}^{m}\left(F_{i}^{MT}\times F_{i}\right)}^{2}$$ (24)

In this way, the group A formed by the fatigue resistant muscle fibers is recruited first, and the group B formed by the less fatigue resistant fibers is recruited later, if needed, which yields a novel muscle recruitment criterion to gather the Henneman’s size principle (Henneman et al., 1965).

### 2.7 Model vs. experimental comparisons

Geometric, force and fatigue muscle parameters were calibrated for each approach implemented in this work as described in the previous sections. Then, the corresponding results were compared with experimental measurements obtained from the strain gauge and the EMG system. Estimated muscle forces were compared with strain gauge readings during MVCs only, because it is assumed that all muscles are fully activated during a maximum effort, thus avoiding the muscle force sharing problem and the uncertainty on the level of effort made by the subject. Whereas, during the dynamic and submaximal efforts EMG measurements were used to compare the estimated relative muscle activations obtained by solving the muscle redundancy problem through optimization to observed EMG activations. Differences between observed and modeled results were qualitatively compared for the 2M, 7M and 14M musculoskeletal models for solving the force sharing problem. Supplementary Appendix SA2 offers an overview of the different configurations implemented in this work which produced the numerous results presented below.

## 3 Results

### 3.1 Static effort

**FIGURE 7:**
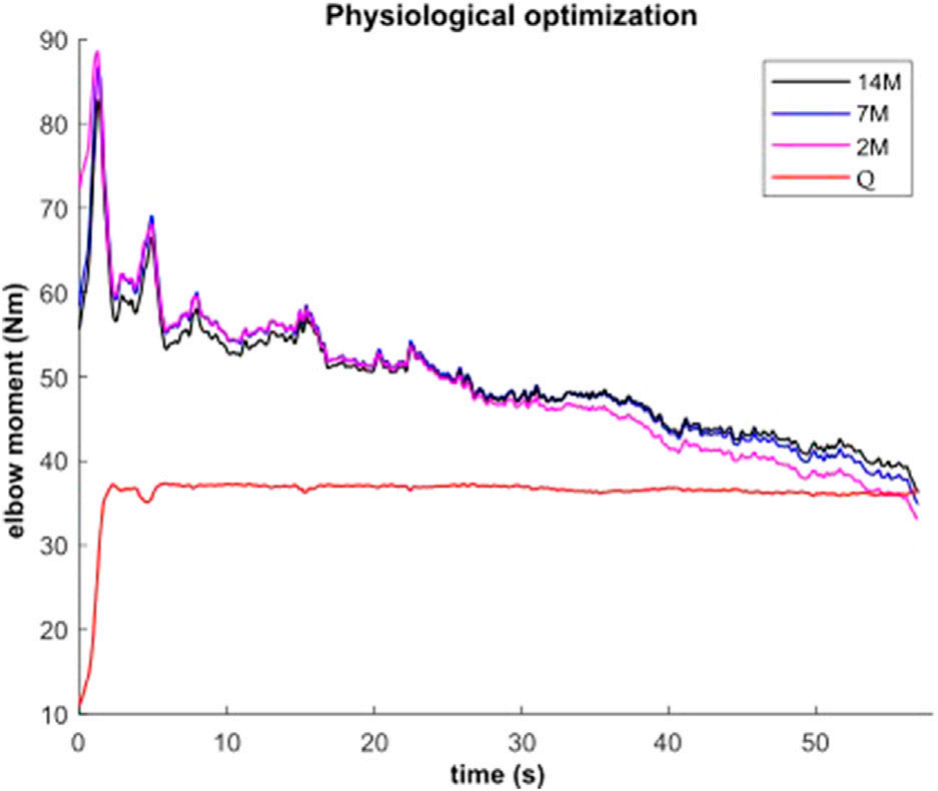
Available maximal elbow flexion moment evolution during static effort using physiological optimization and three muscle models (14M incorporates 2 fiber type groups for 7 muscles; 7M does not consider fiber type; and 2M groups the flexors in one actuator and the extensors in another).

**FIGURE 8:**
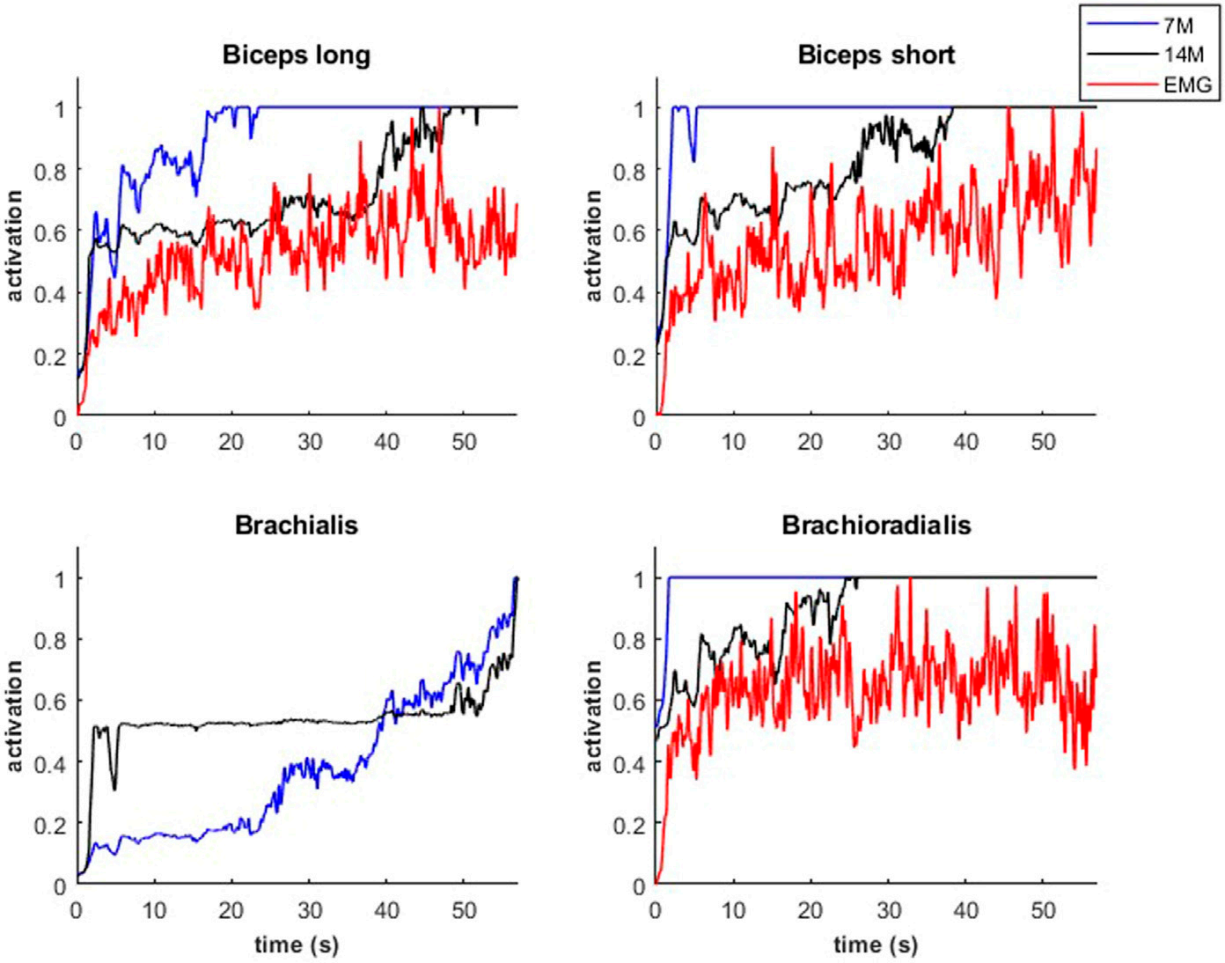
Activations of flexor muscles during the submaximal static effort.

During the submaximal static effort, the subject was able to hold the dumbbell with the arm flexed for 57.0 s (Figure 7, *Q*, in red). The corresponding modeled time course of maximal elbow torque decay for the three musculoskeletal modeling options (14M, 7M, and 2M) based on the physiological optimization for muscle force is shown in Figure 7. The modeled time to failure occurs when the available maximum elbow flexion moment crosses the elbow flexion moment (*Q*, in red) required by the subject to support the dumbbell. All three models were able to predict failure <3 s short of the observed failure (<0.01%–4.4% error). The joint-level actuation (2M, magenta) predicts the earliest failure (54.5 s, 2.5 s early), followed by the classical individual muscle modeling (7M, blue; 56.6 s, 0.4 s early) and the model that considers fiber type dissociation (14M, black; 56.9 s, 0.1 s early). The modeled fiber type force distribution for the elbow flexors for the 14M model confirmed the group A fiber types were recruited prior to the group B, as designed. The net recruitment strategies for the classical individual muscle model (7M, blue) and the fiber type model (14M, black) are shown in Figure 7. Note that differences between the two models are apparent particularly in the activation of brachialis versus the other three flexors. Despite signal filtering, the EMG measurements (Figure 8, red line) obtained during the submaximal static effort were highly variable, with a rapid activation at task initiation and continued increased amplitude along the effort as muscle fatigue occurred. A behavior which appears more similar to the results obtained with the 14M model (black) than the 7M model (blue).

### 3.2 Dynamic effort: Training session

**FIGURE 9:**
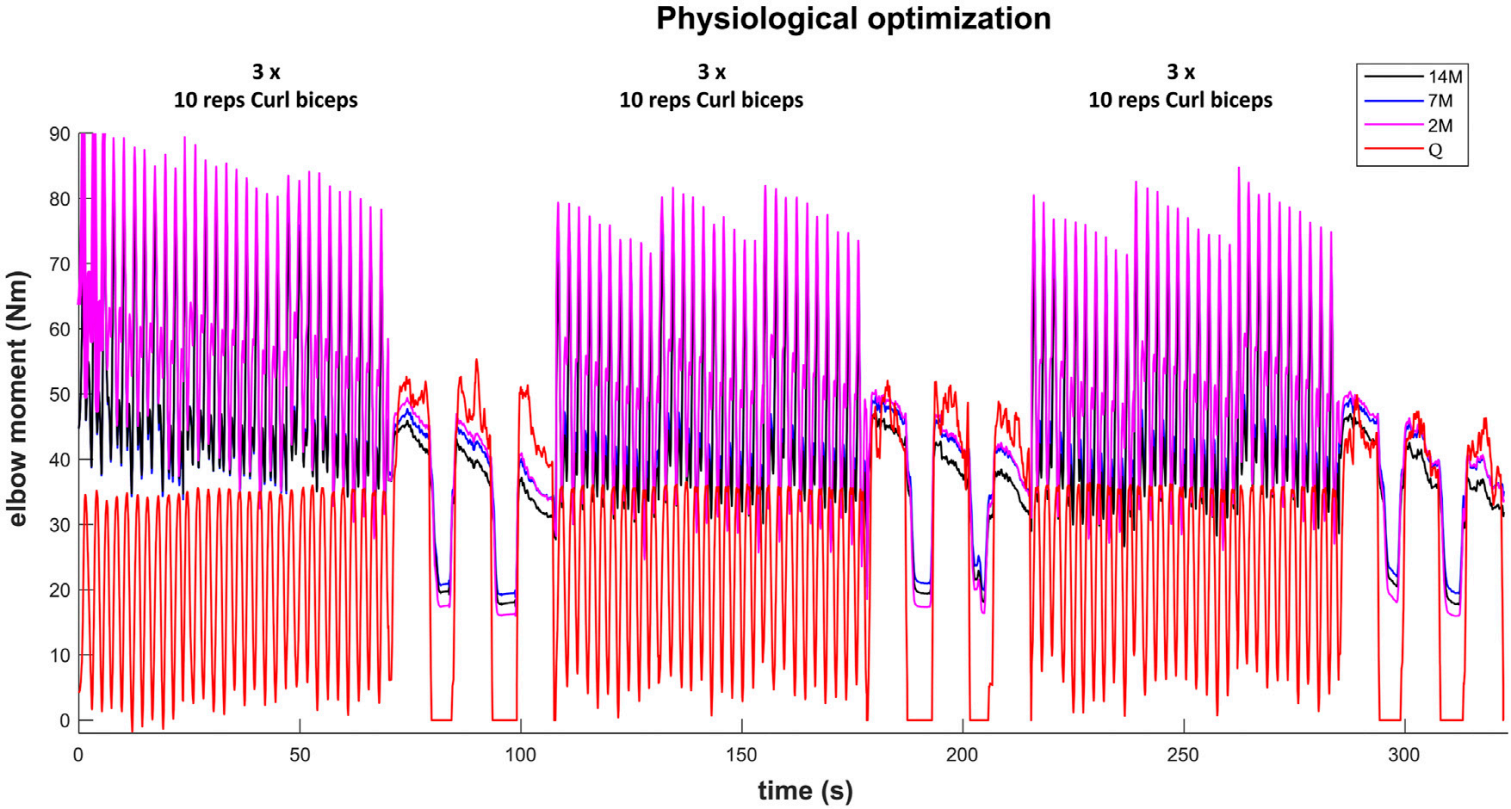
Available maximal elbow flexion moment evolution during the complete training session (note, resting periods are omitted) using three muscular models and physiological optimization.

The results for the dynamic efforts (the weightlifting series and the MVC trials between repetitions) show that, as designed, the available maximal elbow moment decreased during the exercise due to fatigue and that it increased again after the resting periods. However, they underestimated MVC1, MVC2-2 and MVC2-3 and were a close approximation of MVC2-1 and MVC3 relative to the measured flexion torque (see Figure 9). The observed elbow moment was higher for the second MVC than the first, which was not the case for the modeled maximal capabilities. Models 2M and 7M present similar results, while approach 14M presents lower available maximal flexion moments. It must be commented that, during the weightlifting series, after a few series, it was observed that, for some instants, the estimated muscles forces were not able to produce the elbow moment actually made by the subject. Furthermore, to quantify the differences between modeled and observed muscle torque, the root-mean-square error (RMSE) of the three periods of each MVC were calculated, taking as reference the actual exerted flexion moment (see Table 2). The three models showed the highest errors during MVC1, and improved successively for MVC2 and MVC3, reaching a mean RMSE lower than 5 Nm for MVC3. Converse to the static effort, the joint-level 2M model showed the best results, while the 14M yielded the highest errors.

**TABLE 2:** RMSE during each MVC using physiological optimization.

		RMSE maximum elbow moment (Nm)		
		Modeling approach		
		2M	7M	14M
MVC1	MVC1-1	3.10	4.20	6.50
	MVC1-2	5.09	6.07	8.80
	MVC1-3	8.07	8.57	11.16
	Mean MVC1	5.42	6.28	8.82
MVC2	MVC2-1	4.07	3.38	3.18
	MVC2-2	4.06	4.82	7.52
	MVC2-3	4.76	5.55	8.36
	Mean MVC2	4.30	4.58	6.36
MVC3	MVC3-1	5.36	4.34	2.61
	MVC3-2	1.63	1.76	3.86
	MVC3-3	3.43	3.99	6.43
	Mean MVC3	3.47	3.36	4.30
Mean total		**4.40**	**4.74**	**6.49**

The bold values are the mean values.

For some applications, it could be interesting to predict the maximum force that a subject can make after an effort and a resting period. This could be done by focusing on the first elbow moment peak obtained for the three recorded MVC. Table 3 presents the differences between estimated and measured first peak elbow moment obtained with the three muscular models. For the three models, the first peak of MVC1 was underestimated, the first peak of MVC2 was the best predicted one, and the first peak of MVC3 was overestimated. The mean of the absolute error of the three MVC was quite small (lower than 4.6 Nm) and 14M model presented the best results.

**TABLE 3:** First elbow moment peak error using physiological optimization.

	First elbow moment peak error (Nm)		
	Modeling approach		
	2M	7M	14M
MVC1-1	−3.43	−4.67	−6.57
MVC2-1	3.69	2.42	0.13
MVC3-1	6.69	5.41	3.31
Mean of the absolute error	**4.60**	**4.17**	**3.34**

The bold values are the mean values.

**FIGURE 10:**
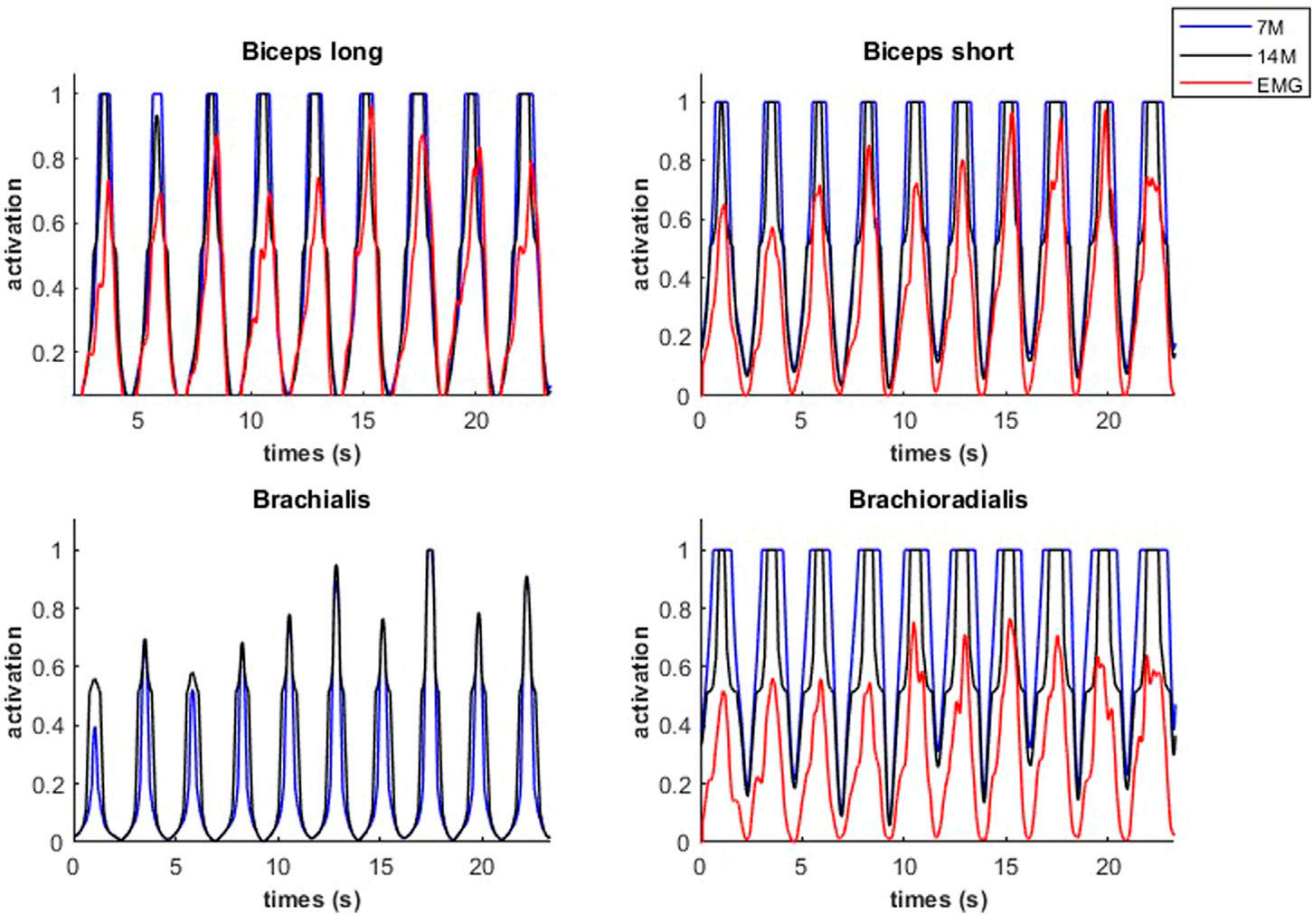
Muscular activations obtained through two muscle models, and EMG signals, during a weightlifting set of 10 bicep curls, using physiological optimization.

Modeled muscle activity for the 7M and 14M models showed similarities but generally exceeded observed EMG measurements (Figure 10) during a weightlifting set.

**FIGURE 11:**
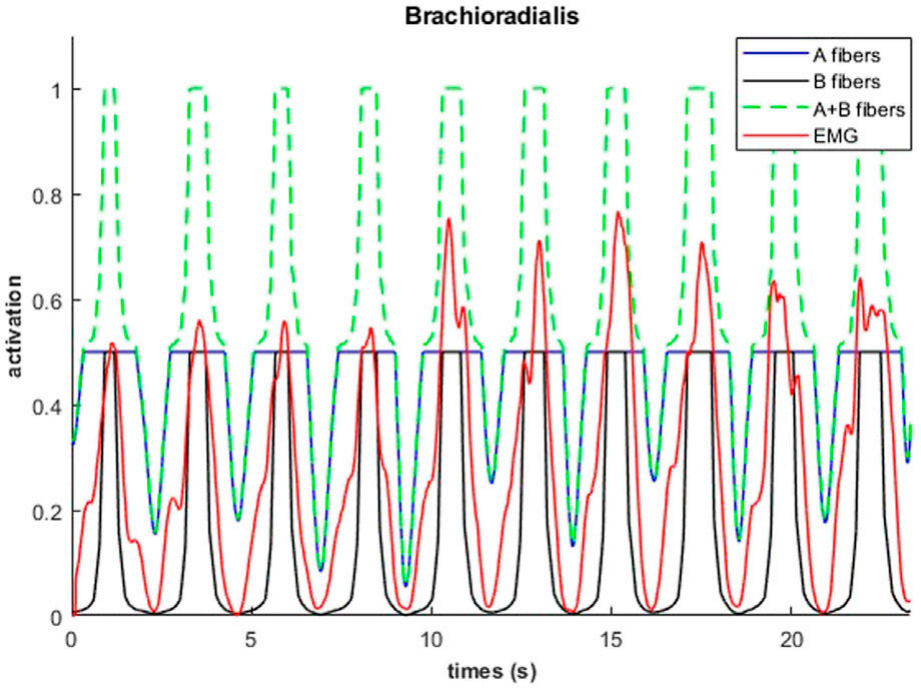
Muscular activations obtained through 14M model (distinction between fibers of type A and B), and EMG signals, during a weightlifting set of 10 repetitions, using physiological optimization.

An example of fiber type specific activation for bicep curls using the 14M model is provided for one muscle in Figure 11. Again, the fatigue-resistant group A units are activated prior to the more fatigable group B units. Interestingly the activations of type B fibers correspond to the increments of the EMG signals.

### 3.3 Fatigue behavior

**FIGURE 12:**
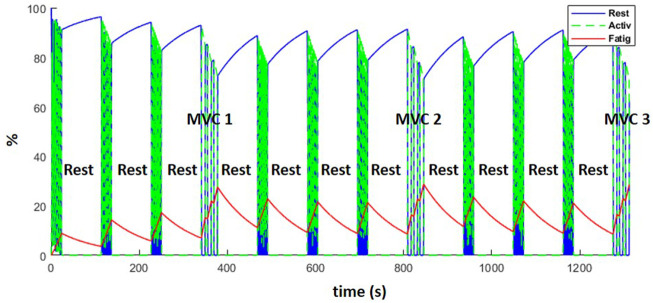
Evolution of the resting (blue), active (green) and fatigue (red) compartments of the biceps long head, during the complete training session (including MVCs every fourth set and resting periods).

Figure 12 represents the evolution of the resting (blue), active (green) and fatigue (red) compartments of the biceps long head, during the complete training session (including the resting periods). Fatigue (which is equal to 100—active plus resting muscle) increased during the exercises and decreased during the resting periods as expected. While recovery was not complete after the rest periods, the MVCs induced more fatigue than the dynamic exercise, such that from the fourth weightlifting set on, force regeneration was higher than its loss.

## 4 Discussion

In this work, multilevel musculoskeletal models for obtaining muscular forces considering fatigue have been assessed relative to experimental measurements for a benchmark case, and the difficulties for achieving accurate subject-specific modeling have been highlighted. Previous studies (Barman et al., 2022), (Pereira et al., 2011), explored the possibility of coupling multibody models and muscular fatigue models for muscle force estimation, but none of them applied the resulting model to a real case. Consequently, they did not address the numerous issues which come up in the calibration of subject-specific parameters, as shown here. We proposed a novel muscle model and a novel force recruitment criterion which reflect the recruitment hierarchy in force generation (Henneman et al., 1965): the muscle fiber-type distinction has been considered and included in the optimization problem.

During static effort, the novel muscle model, 14M, yielded the best results by offering an estimation of the failure instant with an error of less than 1 s. However, all three models (14M, 7M, 2M) yielded good results, which means that the calibration of subject-specific parameters was acceptable. The Law of Parsimony suggests that the simplest model to achieve good results is ideal, thus depending on the accuracy needed for a particular application, any of the three models may be optimal. Using the classical muscle model, 7M, muscles were recruited according to their moment arm only, in order to optimize energy consumption. However, the novel muscle model, 14M, also takes in account a simplified representation of the Henneman’s size principle (Henneman et al., 1965), recruiting fatigue-resistant fibers first (representing type I and IIa fibers collectively). Muscle models with individual muscles (7M and 14M) can yield more accurate joint reactions, bone stresses and energy expenditure estimation (Michaud et al., 2019). Despite noisy EMG signals, the 14M model offered the most similar muscle recruitment strategy of the muscles assessed. Having the EMG measurements of the brachialis would have been very interesting, because its recruitment was the most affected by the method. Unfortunately it is rather difficult to get clean surface signals from this muscle: it is located between biceps and triceps, presents a reduced superficial part, and its proximity to other muscles makes crosstalk inevitable (Jungtäubl et al., 2018).

During the dynamic effort, good results were obtained too, with reasonable errors in MVC estimates whatever the approach used. However, during the dynamic curls, the modeled muscular forces were not as accurate, which provoked constraint violations and muscular overactuation (along with higher fatigue). One explanation for this error may be the mismatch between required elbow moment and flexor muscle moment arms when the forearm got closer to the horizontal position (end of the elbow extension); the flexor moment arms took the lowest values, while the required elbow moment achieved the highest value, leading to a critical situation when using an imperfect model. Consequently, the model and its calibration are crucial aspects. Obviously, if the subject was able to lift the weight during the experiment, the simulation should be able to do so as well. Non-etheless, as illustrated in this study, human modeling and subject-specific calibration are very challenging in biomechanics (Fregly, 2021), since errors can come from many sources (Hicks et al., 2015).

An additional factor that makes calibration and validation challenging is the phenomenon of muscular potentiation. It is essentially the opposite of muscle fatigue where as muscle is first activated, muscle performance is enhanced (Lorenz, 2011; Blazevich and Babault, 2019). Experimental measurements reflected this fact, where the second peak of each MVC was higher than the first, despite fatigue. While the parameters seemed to be well adjusted during the isometric effort, the dynamic effort showed worse results. The effects of the physiological behavior of the muscle, the variations of the moment arm, and differences in activation levels are more relevant during dynamic efforts, thus contributing to the sensitivity and potential inaccuracy of the related parameters. In other studies, muscle parameters are generally adapted to allow muscles to produce the calculated joint torques, adding even a reserve (by overincreasing $F_{0}^{M}$ ), or adding residual actuators to the model (Documentation, 2022). Further, using multiple muscle activation levels, superior model parameter identification was achieved compared to using only one activation level (Frey Law and Shields, 2005), suggesting in many muscle models, parameters do not represent all activation levels equally well.

Another challenging parameter is the estimate of maximum force. If the maximum force is overestimated, the relative intensity to perform a task will be underestimated and accordingly fatigue will be underestimated, and *vice versa*. Therefore, calibration is needed to approximate, as close as possible, the thin force and fatigue limits of the subject (muscles forces have to be able to produce the calculated joint torques without being underactivated) which is more challenging. We observed that the force measurements were also noisy during MVC, which hindered calibration. This may have been due to common variability in achieving voluntary peak force, as well as the attempt to measure elbow peak torque through the involvement of several link lengths (e.g., grip to hold the rope and the wrist). However, the maximum elbow moment, estimated through several methods, did not show significant variation between the three MVCs.

For this reason, the longer MVC (until failure, MVC0-2) was used as reference for the calibration of fatigue parameters. Furthermore, although the actually exerted elbow moment showed a slight decrease from MVC1 to MVC3, likely due to fatigue, the available maximum elbow moment, estimated through several methods, did not reflect this expected force decay along the training session. The compartment fatigue model is a simplified representation of the very complex and dynamic behavior that occurs with muscle fatigue and recovery. As observed with the dynamic bicep curls, in which, even with the MVCs added to the exercise, a stable equilibrium was reached where the model would indicate this level of activity could be maintained indefinitely. Others have also identified this asymptote as a function of the *F* and *R* ratios, that is, for sustained static tasks this was identified as [1/(*F/R* + 1)] ∗ 100% (Frey-Law et al., 2012). For cyclical dynamic tasks with rest intervals, this asymptote is not quite so simple, yet still occurs at the point at which recovery and fatigue balance. However, this is not realistic for most individuals. To address this, the fatigue model could be modified for this application, either by changing the model parameter values, adding in a central fatigue representation [referred to as “brain effort” by (Xia and Frey Law, 2008)], or by adding a fourth compartment off of the fatigued state which could be called “long-term fatigue state,” so as to generate a reversible fatigue only after a long resting period.

## 5 Conclusion

In conclusion, this study evaluates different alternatives to include a muscle fatigue model in the muscle recruitment problem, for short-term high-intensity exercises, by comparing their results with those from experimental measurements within a benchmark case. All the methods yield reasonable estimations, but the complexity of obtaining accurate subject-specific human models (sometimes hidden in other works because parameters are generally adapted to allow muscles to produce the calculated joint torques) is highlighted in this study. The Law of Parsimony suggests that the simplest model (2M) to achieve good results is ideal, thus depending on the accuracy needed for a particular application, any of the three models may be optimal. However, muscle models with individual muscles (7M and 14M) can yield more accurate joint reactions and bone stresses. The proposed novel muscle modeling and force recruitment criterion (14M), which consider the muscular fiber-type distinction, show interesting preliminary results, but model calibration needs to be improved to fully validate this approach.

## Data Availability

The raw data supporting the conclusion of this article will be made available by the authors, without undue reservation.

## Supplementary material

SUPPLEMENTARY FILE S1 Model specific details.

SUPPLEMENTARY FILE S2 Study Conceptual Design.

## Nomenclature

### List of symbols

α: pennation angle
a: muscle activation
$C_{f}$: objective function of the load-sharing problem
φ: elbow flexion angle
$f_{l}$: dimensionless force-length relationship
$f_{v}$: dimensionless force-velocity relationship
$f_{PE}$: dimensionless force-length relationship
***F***: fatigue coefficient
$F_{A}$: group A fatigue coefficient
$F_{B}$: group B fatigue coefficient
$F_{CE}^{M}$: force exerted by the contractile element
$F_{CE,\mathrm{Max}}^{M}$: maximum force allowed by the contractile element
$F_{PE}^{M}$: force exerted by the passive element
$F_{i}^{\mathrm{Min}}$: instantaneous minimum allowed force for muscle *i*
$F_{i}^{\mathrm{Max}}$: instantaneous maximum allowed force for muscle *i*
$F_{Phys,Ext}^{\mathrm{Min}}$: minimum allowed force by the extensor muscles
$F_{Phys,Flex}^{\mathrm{Max}}$: maximum allowed force by the flexor muscles
$F_{0}^{M}$: maximum isometric force
$F_{0}^{M*}$: scaled maximum isometric forces
$F_{0,A}^{M}$: group A maximum isometric force
$F_{0,B}^{M}$: group B maximum isometric force
$F^{MT}$: the vector of individual muscle forces
J: Jacobian whose transpose projects the muscle forces into the joint drive torques space
$J_{Flex}$: Jacobians whose transpose projects the muscle forces into the joint drive torques space of flexor muscles
$J_{Ext}$: Jacobians whose transpose projects the muscle forces into the joint drive torques space of extensor muscles
$k_{1}$: moment arms scale factor
$k_{2}$: maximum isometric force scale factor
$l_{0}^{M}$: optimal muscle fiber length
$l_{S}^{T}$: tendon slack length
*M*_*a*_: percentage of motor units activated
*M*_*f*_: percentage of motor units fatigued
*M*_r_: percentage of motor units resting
Q: vector of joint torques obtained by inverse dynamics
$Q_{\mathrm{Max},Flex}$: maximum inverse-dynamics elbow joint flexion moment
$Q_{\mathrm{Max},Flex}^{*}$: maximum inverse-dynamics elbow joint flexion moment taking into account the passive moment generated by the extensor muscles
***r***: rest multiplier to augment recovery during rest
$r_{A}$: group A rest multiplier to augment recovery during rest
$r_{B}$: group B rest multiplier to augment recovery during rest
*R*: recovery coefficient
$R_{A}$: group A recovery coefficient
$R_{B}$: group B recovery coefficient
$v^{M}$: contractile element velocity
$v^{MT}$: musculotendon velocity

### Acronyms

2M: joint level analysis, one flexor and one extensor actuator
3CC: three-compartment controller model
3CCr: three-compartment controller model with additional rest recovery parameter
7M: 7-muscle arm model
14M: 14-muscle arm model
DAS: dynamic arm simulator
EMG: electromyography
MU: motor units
MVC: isometric maximum voluntary contraction
PCSA: physiologic cross sectional area
TL: target load
