# Supplementary figures and images for: Applying a muscle fatigue model when optimizing load-sharing between muscles for short-duration high-intensity exercise: A preliminary study
Source: Front Physiol. 2023 Apr 24;14:1167748. doi: 10.3389/fphys.2023.1167748 (PMC10165736; doi:10.3389/fphys.2023.1167748)

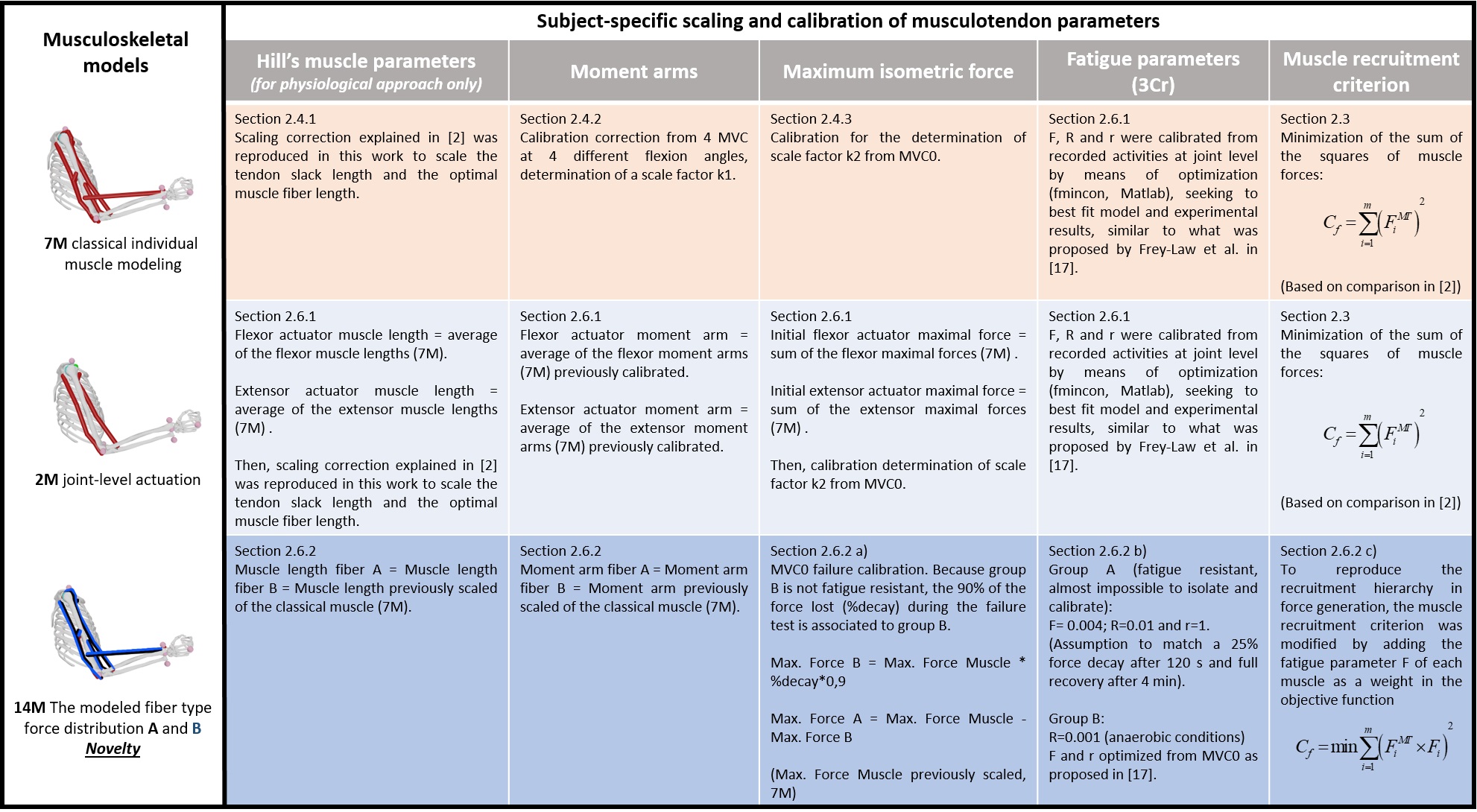

Supplement: Supplementary file 1 [file Image1.JPEG]

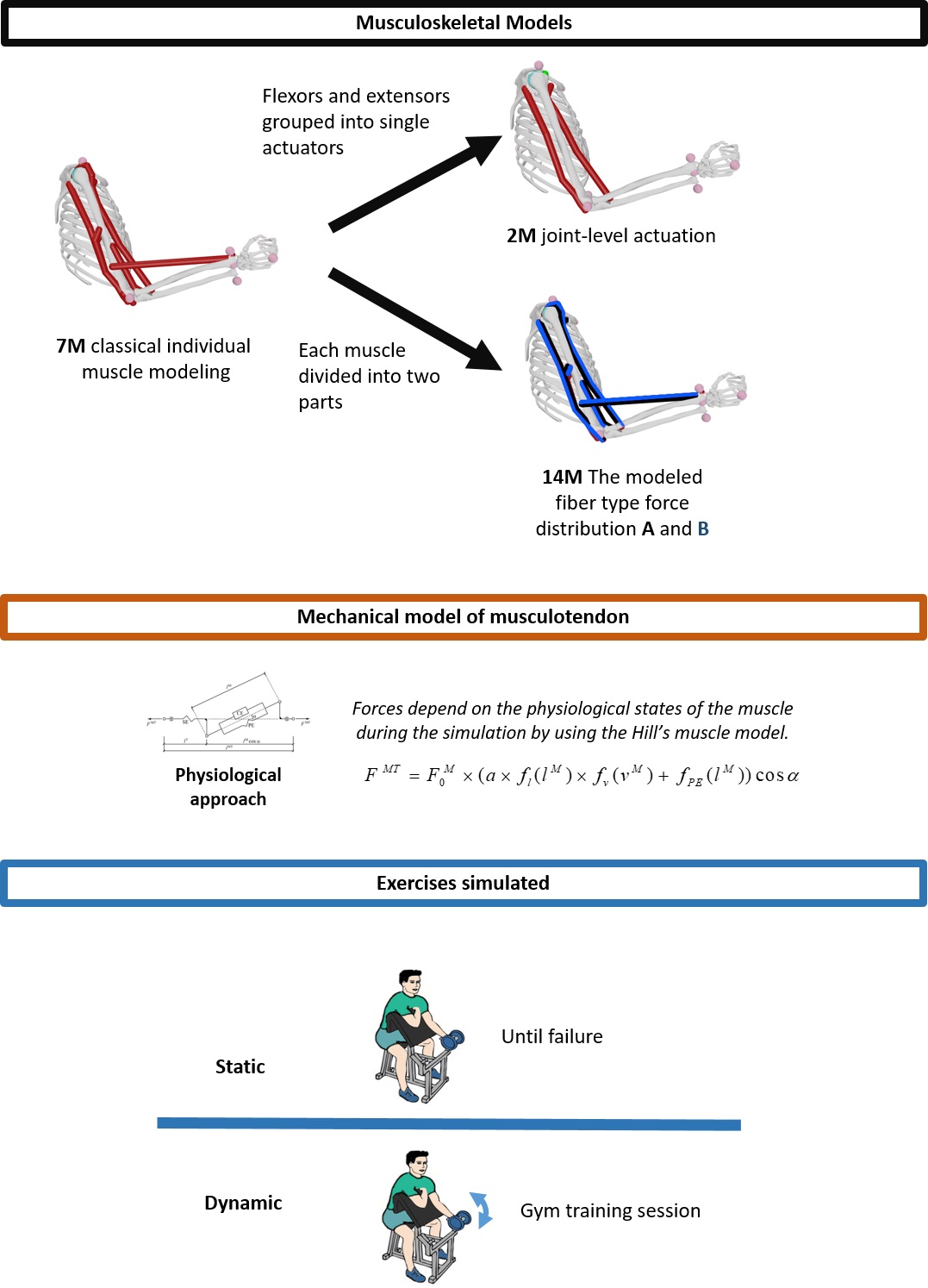

Supplement: Supplementary file 2 [file Image2.JPEG]
